# The multigenerational effects of adolescent motherhood on school readiness: A population-based retrospective cohort study

**DOI:** 10.1371/journal.pone.0211284

**Published:** 2019-02-06

**Authors:** Elizabeth Wall-Wieler, Janelle Boram Lee, Nathan Nickel, Leslie Leon Roos

**Affiliations:** 1 Stanford University, Stanford, California, United States of America; 2 Manitoba Centre for Health Policy, Winnipeg, Manitoba, Canada; 3 Community Health Sciences, University of Manitoba, Winnipeg, Manitoba, Canada; International Telematic University Uninettuno, ITALY

## Abstract

**Background:**

Children born to adolescent mothers generally perform more poorly on school readiness assessments than their peers born to adult mothers. It is unknown, however, whether this relationship extends to the grandchildren of these adolescent mothers. This paper examines the multi-generational outcomes associated with adolescent motherhood by testing whether the grandchildren of adolescent mothers also have lower school readiness scores than their peers; we further assessed if this relationship was moderated by whether the child’s mother was an adolescent mother.

**Methods:**

We used population-based data to conduct the retrospective cohort study of children born in Manitoba, Canada, 2000–2009, whose mothers were born 1979–1997 (*n* = 11,326). Overall school readiness and readiness on five domains of development were analyzed using logistic regression models.

**Results:**

Compared with children whose mothers and grandmothers were both ≥ 20 at the birth of their first child, those born to grandmothers who were < 20 and mothers who were ≥ 20 years old at the birth of their first child had 39% greater odds of being not ready for school (95% CI: 1.22–1.60). Children whose grandmothers were ≥ 20 and mothers were < 20 at the birth of their first child had 25% greater odds of being not ready for school (95% CI: 1.11–1.41), and children born to grandmothers and mothers who were both <20 at the birth of their first child had 35% greater odds of being not ready for school (95% CI: 1.18–1.54).

**Conclusions:**

These findings suggest a multigenerational effect of adolescent motherhood on school readiness.

## Introduction

While children from different backgrounds have different resources and opportunities, one of the tenets of the public education is to reduce the inequalities of poverty and circumstance by providing all children with an equal opportunity of success. However, children enter school at various degrees of readiness, and readiness at school entry has significant effects on how children perform throughout their education.[1–3] Specific characteristics associated with being less ready for school include living in poverty, low levels of parental education, family and neighborhood instability, and having mothers who were very young when they became parents.[1,4–7] Pre-kindergarten programs–such as Head Start–were introduced to address this gap in school readiness and have recorded some success, particularly among children with low cognitive ability and those whose parents had low levels of education.[8] However, differences in school readiness persist, and the risks associated with starting school at a disadvantage can accumulate over the life course.

Children born to adolescent mothers have been identified as being less ready for school and having poorer educational outcomes. For example, Canadian children of adolescent mothers average 1.6 fewer years of education; 19% of children born to adolescent mothers in the UK continue after compulsory school, compared with 31% of children not born to adolescent mothers.[9,10] These findings have been attributed to several mechanisms. First, adolescent mothers tend to have lower educational attainment themselves. Some adolescents become pregnant after dropping out of high school, while others drop out of high school when they become pregnant.[11,12] Maternal education level strongly influences children’s cognitive and behavioral outcomes; mothers with higher education tend to have greater expectations of their children and to provide more cognitively stimulating home environments.[13,14] Second, adolescent mothers often have fewer monetary and non-monetary resources, as they are more likely to be single and to be living in poverty.[15] Lack of monetary resources results in fewer educational materials in the home, less educational enrichment outside of school, and a greater likelihood of experiencing food insecurity.[16,17] Lone mothers also have fewer nonmonetary resources, such as time and social supports, making it more difficult to participate in their child’s schooling.[17,18]

Although the rate of adolescents becoming pregnant and having children is declining among several population groups, the rates of adolescent motherhood vary significantly across jurisdictions and by socioeconomic status.[19] Adolescent pregnancy rates are particularly high among those with a family history of adolescent childbearing.[20–23] This intergenerational transmission of adolescent childbearing has been attributed both to the transmission of resources and to the transmission of social and cultural norms.[24] Adolescent mothers tend to raise their children in more socioeconomically disadvantaged environments, which in turn contributes to higher rates of adolescent pregnancy among their daughters.[25,26] Thus, the resources and norms passed on to adolescent mothers from their families may be a mechanism for lower levels of school readiness in their offspring.

Several pathways have been identified as contributing to the accumulation of disadvantage across generations for children born to adolescent mothers. The most severe intergenerational consequences have been attributed to lack of material and economic resources (i.e. poverty).[27] Adolescent mothers are much more likely to live in poverty; being raised in poverty results in children in the next generation to have lower cognitive ability.[28,29] In addition to the impact of poverty through generations, cognitive ability and school readiness can also be affected by genetics and by cultural and social norms. Differences in spatial, verbal, and memory ability have all been attributed in part to genetic influences. Maternal behaviors during pregnancy—smoking, substance use, and prenatal care–are strongly affected by social and cultural norms and can affect school readiness.[30] Intergenerational correlations in birth weight–which is linked to childhood development–have also been identified.[31,32] The degree to which these pathways contribute to the multigenerational effects of adolescent motherhood on school readiness is not known. However, the sum of these potential pathways are very likely to have some effect.

This study investigates the possibility of a multigenerational effect of adolescent motherhood on school readiness. The relationship between adolescent motherhood and school readiness among her grandchildren is examined both for overall school readiness and for readiness on five specific developmental domains (physical health and wellbeing, social competence, emotional maturity, language and cognitive skills, and communication skills and general knowledge). We also compare children with two generations of adolescent motherhood with those experiencing only one generation of adolescent motherhood. This research provides novel insight into the multigenerational effects of adolescent motherhood on school readiness; such studies have not been done previously as they require a large cohort of individuals with longitudinal data available across three generations. The unique linkable administrative data available in Manitoba, Canada are well suited to studying this relationship.

## Materials and methods

### Data and setting

Manitoba is a central Canadian province; at the time of the 2011 census, there were approximately 1.2 million residents.[33] Approximately 30.2% of Manitoba children were vulnerable in at least one area of development at age five; this was higher than the Canadian average of 26% and the Australian average of 22%. In 2010, adolescent pregnancy rates in Canada were 28.2 per 1,000; in Manitoba, the rate was 48.7 per 1,000. Manitoba adolescent pregnancy rates in 2010 were slightly lower than those in the United States (57.4 per 1,000), and in England and Wales (54.6 per 1,000), but higher than rates seen in Australia (34 per 1,000).[34–36]

The Manitoba Population Research Data Repository contains province-wide, routinely collected individual data for each resident.[37] Our research linked data from the population registry with individual-level information from hospital discharge abstracts, Early Development Instrument (EDI) data (administered by the Healthy Child Manitoba Office), and the Canadian Census. An anonymized personal health number allowed linkage of these de-identified datasets. Information on linkage methods, confidentiality, privacy, and validity is fully documented, [38,39]

### Variables

#### School readiness

School readiness is measured by the Early Development Instrument (EDI), a 103-item questionnaire administered by kindergarten teachers in their classrooms in the second half of the school year.[40] This questionnaire assesses five areas of development in kindergarten using binary and Likert-scale items–physical health and well-being, social competence, emotional maturity, language and cognitive development, and communication skills and general knowledge.[41] To ensure accurate, consistent interpretation of results, teachers who administer the EDI attend training/information sessions; EDI scores show acceptable inter-rater reliability and high internal consistency.[41] No systematic measurement differences have been found among cultural groups.[42] Results are comparable across contexts, and consistent relationships have been found with other developmental tests.[43,44]

Each area of development includes a range of characteristics: 1) the physical health and well-being domain includes measures on the child’s physical readiness for the school day, physical independence, and gross and fine motor skills; 2) the social competence domain includes responsibility and respect, approaches to learning, and readiness to explore new things; 3) the emotional maturity domain includes measures of pro-social and helpful behavior, anxious and fearful behavior, and hyperactivity and inattention; 4) the language and cognitive development domain includes basic literacy, interest in literacy and numeracy, advanced literacy, and basic numeracy; 5) the communication skills and general knowledge domain includes skill to communicate effectively, symbolic use of language, and age-appropriate knowledge about the world.[45] The scores for each domain are converted to a score from 1 (most vulnerable) to 10 (most ready), and children falling in the lowest 10 percentile in a domain are considered ‘not ready’ or ‘vulnerable’.[42] Children who are vulnerable on one or more domains of the EDI have significantly worse reading, writing, and math scores in grade 3.[2]

The current study examined readiness based on each of five developmental areas and an overall score of school readiness. School readiness is defined as ready and not ready; based on national norms, children scoring in the lowest 10 percent in each domain are considered ‘not ready’.[41] The overall measure of not being ready for school is defined as being not ready in one or more developmental areas.

#### Adolescent motherhood

Adolescent motherhood is defined as a mother who gave birth to her first child before she turned 20.[46] The age 20 cut-off has been adopted by the World Health, and used consistently in studies of adolescent motherhood.[47–49] Thus, any children born to mothers whose first child was born before age 20 are noted as having an adolescent mother, even if their mother was 20 or older when they were born. Age at first birth for mothers and grandmothers is identified through the Manitoba Insurance Registry.

#### Covariates

For both the child and the mother, we adjusted for their birth year, location (urban, rural) and income quintile (1 = *lowest income* to 5 = *highest income*) of the neighborhood they lived in at birth. We also adjusted for the characteristics and health of the child: sex, birth order, health at birth (whether the child was preterm (<37 weeks) or had low birth weight (<2500 g), mental and physical health before age five, and parental receipt of Employment and Income Assistance (EIA–analogous to welfare) before age 5. To account for the associations of early childhood mental health with lower school attainment, we included two mental health conditions seen in early childhood—attention-deficit/hyperactivity disorder (ADHD) and Conduct Disorder.[3,50] Physical health was measured by hospitalizations for injury and diagnoses of asthma. These measures were selected as injuries are the leading cause of death in children, while asthma is the most common chronic condition in childhood.[50] Definitions of childhood covariates between birth and age 5 can be found in S1 File and S1 Table.

### Cohort formation

The Manitoba government began administration of the EDI in the 2005–2006 school year. Six EDI cycles were available: 2005–06, 2006–07, 2008–09, 2010–11, 2012–13, and 2014–15. This research included individuals who had a valid EDI score (completed between 5^th^ and 7^th^ birthdays), lived in Manitoba from birth until completion of their EDI, were not identified as having special needs, whose mothers were born in or after 1979, and had no missing values on covariates (*n* = 14,298). Mothers born before April 1, 1979 were excluded as information on grandmother’s age at first birth is not available before this date. One child was randomly selected for mothers having more than one child in the cohort using simple random sampling, reducing the cohort to 11,326 children. S1 Fig in the Supplemental Materials details the population cohort selection process.

### Statistical analysis

Three sets of logistic regression models obtained unadjusted and adjusted odds ratios (aORs) for school readiness in the cohort. The first set of models examined the association between mother’s age at first birth and school readiness. This analysis helps assess the extent to which Manitoba results compare with other on adolescent motherhood and school readiness. The second set of models examined the association between grandmother’s age at first birth and school readiness, providing insight into the multigenerational effects of adolescent motherhood. The final models considered the association for both mothers’ and grandmothers’ age at first birth and school readiness. For this model, a categorical variable was created: neither mother nor grandmother were adolescent mothers, only mother was an adolescent mother, only grandmother was an adolescent mother, and both mother and grandmother were adolescent mothers. Data management, programming, and analyses were performed using SAS version 9.4.[51]

This study was approved by the Health Research Ethics Board at the University of Manitoba (#H2013:164) and the Health Information Privacy Commission at Manitoba Health, Seniors and Active Living (#2013/2014-04). Using de-identified administrative data files did not require informed consent from participants.

## Results

The study cohort included all mothers born in Manitoba between 1979 and 1997 with children born after 2000. This resulted in our cohort having many young mothers Fourty-six percent of the children had mothers less than 20 at the birth of their first child, and 30 percent of the children had grandmothers younger than 20 at the birth of their first child (Table 1).

**Table 1:** Description of Cohort (n = 11,326).

Covariates	n (%)
**Grandmother Variables**	
Average Age at First Birth (Range)	23.05 (13.75–46.69)
Age at First Birth	
<20	3,391 (29.94)
20+	7,935 (70.03)
**Mother Variables**	
Location of Neighborhood at Birth	
Urban	5,095 (44.98)
Rural	6,231 (55.02)
Income Quintile of Neighborhood at Birth	
1 (Lowest)	3,686 (32.54)
2	2,398 (21.17)
3	1,955 (17.26)
4	1,854 (16.37)
5 (Highest)	1,433 (12.65)
Average Age at First Birth (Range)	20.90 (12.61–30.52)
Age at First Birth	
<20	5,179 (45.72)
20+	6,147 (54.27)
Birth Year	
1979–1984	7,341 (64.82)
1985–1991	3,792 (33.48)
1992–1997	193 (1.70)
**Child Variables**	
*At Birth*	
Location of Neighborhood	
Urban	5,807 (51.27)
Rural	5,519 (48.73)
Income Quintile of Neighborhood	
1 (Lowest)	3,614 (31.91)
2	2,507 (22.13)
3	2,280 (20.13)
4	1,705 (15.05)
5 (Highest)	1,220 (10.77)
Year	
2000–2003	2,548 (22.50)
2004–2007	4,715 (41.63)
2008–2010	4,063 (35.87)
Sex	
Male	5,683 (50.18)
Female	49.82)
Birth Order	
1	6,855 (60.52)
2	2,992 (26.42)
3	968 (8.55)
4+	511 (4.51)
Low Birth Weight	669 (5.91)
Preterm	924 (8.16)
*Between Birth and Age 5*	
ADHD Diagnosis	234 (2.07)
Conduct Disorder Diagnosis	270 (2.38)
Hospitalization for Injury	128 (1.13)
Asthma Diagnosis	2,453 (21.66)
Parent(s) Received Welfare	4,379 (38.66)

### Mother’s age at first birth

The initial analysis examined the relationship between mother’s age at first birth and school readiness among children. Among children of adolescent mothers, 43.1% were not ready for school; 26.4% of children of non-adolescent mothers were not ready for school (Table 2). When adjusting for all covariates, the odds of being not ready for school were significantly higher overall (aOR = 1.17), and on the specific domains physical well-being (aOR = 1.17), social competence (aOR = 1.18), and language and cognitive development (aOR = 1.21). Odds ratios associated with all adjustment covariates can be found in S2 Table in the Supplemental Materials.

**Table 2:** Frequencies, unadjusted, and adjusted odds ratios of school readiness by Mother’s Adolescent Motherhood Status.

Not Ready for School	Mother was an Adolescent Mother (n = 5,179)	Mother was not an Adolescent Mother (n = 6,147)	Unadjusted OR (95% CI)	Adjusted^a^ OR (95% CI)
	n (%)	n (%)		
Overall	2,232 (43.10)	1,622 (26.39)	2.11 (1.95, 2.29)	1.17 (1.05, 1.31)
Physical Well-Being	1,103 (21.30)	727 (11.83)	2.01 (1.82, 2.23)	1.17 (1.02, 1.34)
Social Competence	1,030 (19.89)	697 (11.34)	1.94 (1.75, 2.15)	1.18 (1.02, 1.35)
Communication and General Knowledge	784 (15.14)	515 (8.38)	1.95 (1.73, 2.20)	0.99 (0.85, 1.16)
Emotional Maturity	912 (17.61)	702 (11.42)	1.66 (1.49, 1.84)	1.12 (0.97, 1.30)
Language and Cognitive Development	1,084 (20.93)	613 (9.97)	2.39 (2.15, 2.66)	1.21 (1.05, 1.39)

^a^ Adjusted for income quintile and location (urban/rural) of neighborhood at birth of mother, mother’s birth year, income quintile and location (urban/rural) of neighborhood at birth of child, child’s birth year, child’s sex, child’s birth order, child’s birth weight, child’s gestational age, child diagnoses before 5^th^ birthday: ADHD, conduct disorder, asthma, injury hospitalization, parent(s) received welfare

### Grandmother’s age at first birth

The second set of analyses examined, the relationship between grandmother’s age at first birth and her grandchild’s school readiness. A greater percentage of children whose grandmothers had been adolescent mothers were not ready for school (36%) than those children whose grandmothers were 20 or older when their first child was born (31%) (Table 3). After adjusting for all covariates, the odds of being not ready for school were significantly higher for children whose grandmothers had been adolescent mothers (aOR = 1.21); this relationship held for all domains. S3 Table in the Supplemental Materials presents the odds ratios associated with all adjustment covariates.

**Table 3:** Frequencies, unadjusted, and adjusted odds ratios of school readiness by Grandmother’s Adolescent Motherhood Status.

Not Ready for School	Grandmother was an Adolescent Mother (n = 3,391)	Grandmother was not an Adolescent Mother (n = 7,935)	Unadjusted OR (95% CI)	Adjusted^a^ OR (95% CI)
	n (%)	n (%)		
Overall	1404 (36.43)	2450 (30.88)	1.58 (1.46, 1.72)	1.21 (1.11, 1.33)
Physical Well-Being	704 (20.76)	1126 (14.19)	1.58 (1.43, 1.76)	1.23 (1.10, 1.37)
Social Competence	652 (19.23)	1075 (13.55)	1.52 (1.37, 1.69)	1.21 (1.08, 1.36)
Communication and General Knowledge	501 (14.77)	798 (10.06)	1.55 (1.38, 1.75)	1.17 (1.03, 1.32)
Emotional Maturity	571 (16.84)	1043 (13.14)	1.34 (1.20, 1.50)	1.12 (1.00, 1.27)
Language and Cognitive Development	662 (19.52)	1035 (13.04)	1.62 (1.45, 1.80)	1.17 (1.05, 1.31)

^a^ Adjusted for income quintile and location (urban/rural) of neighborhood at birth of mother, mother’s birth year, income quintile and location (urban/rural) of neighborhood at birth of child, child’s birth year, child’s sex, child’s birth order, child’s birth weight, child’s gestational age, child diagnoses before 5^th^ birthday: ADHD, conduct disorder, asthma, injury hospitalization, parent(s) received welfare

### Intergenerational adolescent motherhood

Of the 5,179 children whose mothers had their first child before age 20, 2,062 (39.8%) also had grandmothers who had their first child before age 20. Of the 6,147 children whose mothers had their first child on or after their 20^th^ birthday, only 1,329 (21.6%) had a grandmother who was younger than 20 at the birth of her first child. Overall and for each domain, school readiness was highest among children whose mother and grandmother were not adolescent mothers (Table 4). Having either only a grandmother or only a mother who was an adolescent mother decreased rates of school readiness; the highest percent of children not ready for school was found when both mother and grandmother had been adolescent mothers.

**Table 4:** Frequency and percent of children not ready for school by Mother and Grandmother’s Adolescent Motherhood Status.

Not Ready for School	Neither Mother nor Grandmother (n = 4,818)	Grandmother Only (n = 1,329)	Mother Only (n = 3,117)	Mother and Grandmother (n = 2,062)
	n (%)	n (%)	n (%)	n (%)
Overall	1168 (24.24)	454 (34.16)	1282 (41.13)	2062 (46.07)
Physical Well-Being	509 (10.56)	218 (16.40)	617 (19.79)	486 (23.57)
Social Competence	487 (10.11)	210 (15.80)	588 (18.86)	442 (21.44)
Communication and General Knowledge	354 (7.35)	161 (12.11)	444 (14.24)	340 (16.49)
Emotional Maturity	512 (10.63)	190 (14.30)	531 (17.04)	381 (18.48)
Language and Cognitive Development	422 (8.76)	191 (14.37)	613 (19.67)	471 (22.84)

The final analyses further examined intergenerational adolescent motherhood and school readiness. For each outcome except the communication and general knowledge domain and the emotional maturity domain, if one or both generations (mother/grandmother) had been adolescent mothers, the odds of the child not being ready for school were significantly higher than if neither generation had been an adolescent mother (Table 5). The unadjusted model showed children having adolescent mothers were less likely to be ready for school than those whose grandmothers had been adolescent mothers. Two generations of adolescent motherhood resulted in greater odds of not being ready for school than one generation of adolescent motherhood. After adjustment, the odds of not being ready for school were statistically similar for children with one generation of adolescent motherhood (either grandmother or mother) and two generations of adolescent motherhood. Odds ratios associated with all adjustment covariates can be found in S4 Table in the Supplemental Materials. While many of the adjustment covariates were statistically significant, the sex of the child, and whether the child had an ADHD diagnosis before age 5 consistently led to adjusted odds ratios of over 2 (S2, S3 and S4 Tables).

**Table 5:** Results of unadjusted and adjusted logistic regression models for school readiness by Intergenerational Adolescent Motherhood Status (n = 11,326).

School Readiness Domain	Unadjusted OR (95% CI)	Adjusted^a^ OR (95% CI)
**Overall**		
‘Grandmother Only’ vs ‘Neither’	1.62 (1.42, 1.85)	1.39 (1.22, 1.60)
‘Mother Only’ vs ‘Neither’	2.18 (1.98, 2.41)	1.25 (1.11, 1.41)
‘Mother and Grandmother’ vs ‘Neither’	2.67 (2.39, 2.98)	1.35 (1.18, 1.54)
‘Mother Only’ vs ‘Grandmother Only’	1.35 (1.18, 1.54)	0.90 (0.77, 1.05)
‘Mother and Grandmother’ vs ‘Grandmother Only’	1.65 (1.43, 1.90)	0.97 (0.82, 1.14)
‘Mother and Grandmother’ vs ‘Mother Only’	1.22 (1.09, 1.37)	1.07 (0.96, 1.21)
**Physical Well-Being**		
‘Grandmother Only’ vs ‘Neither’	1.66 (1.40, 1.97)	1.41 (1.18, 1.68)
‘Mother Only’ vs ‘Neither’	2.09 (1.84, 2.37)	1.24 (1.06, 1.45)
‘Mother and Grandmother’ vs ‘Neither’	2.61 (2.28, 2.99)	1.38 (1.17, 1.64)
‘Mother Only’ vs ‘Grandmother Only’	1.26 (1.06, 1.49)	0.88 (0.73, 1.07)
‘Mother and Grandmother’ vs ‘Grandmother Only’	1.57 (1.32, 1.88)	0.98 (0.80, 1.20)
‘Mother and Grandmother’ vs ‘Mother Only’	1.25 (1.09, 1.43)	1.11 (0.97, 1.28)
**Social Competence**		
‘Grandmother Only’ vs ‘Neither’	1.67 (1.40, 1.99)	1.48 (1.23, 1.77)
‘Mother Only’ vs ‘Neither’	2.07 (1.82, 2.35)	1.29 (1.10, 1.52)
‘Mother and Grandmother’ vs ‘Neither’	2.43 (2.11, 2.79)	1.36 (1.15, 1.63)
‘Mother Only’ vs ‘Grandmother Only’	1.24 (1.04, 1.47)	0.88 (0.73, 1.07)
‘Mother and Grandmother’ vs ‘Grandmother Only’	1.45 (1.21, 1.74)	0.98 (0.80, 1.20)
‘Mother and Grandmother’ vs ‘Mother Only’	1.17 (1.02, 1.35)	1.05 (0.91, 1.22)
**Communication and General Knowledge**		
‘Grandmother Only’ vs ‘Neither’	1.74 (1.43, 2.12)	1.46 (1.19, 1.78)
‘Mother Only’ vs ‘Neither’	2.10 (1.81, 2.43)	1.10 (0.92, 1.32)
‘Mother and Grandmother’ vs ‘Neither’	2.49 (2.12, 2.92)	1.13 (0.93, 1.37)
‘Mother Only’ vs ‘Grandmother Only’	1.20 (0.99, 1.460	0.76 (0.61, 0.94)
‘Mother and Grandmother’ vs ‘Grandmother Only’	1.43 (1.17, 1.75)	0.78 (0.62, 0.97)
‘Mother and Grandmother’ vs ‘Mother Only’	1.19 (1.02, 1.39)	1.03 (0.87, 1.20)
**Emotional Maturity**		
‘Grandmother Only’ vs ‘Neither’	1.40 (1.17, 1.68)	1.28 (1.07, 1.55)
‘Mother Only’ vs ‘Neither’	1.73 (1.52, 1.97)	1.20 (1.02, 1.41)
‘Mother and Grandmother’ vs ‘Neither’	1.91 (1.65, 2.20)	1.22 (1.02, 1.46)
‘Mother Only’ vs ‘Grandmother Only’	1.23 (1.03, 1.47)	0.93 (0.76, 1.14)
‘Mother and Grandmother’ vs ‘Grandmother Only’	1.36 (1.12, 1.64)	0.95 (0.77, 1.18)
‘Mother and Grandmother’ vs ‘Mother Only’	1.10 (0.95, 1.28)	1.02 (0.88, 1.18)
**Language and Cognitive Development**		
‘Grandmother Only’ vs ‘Neither’	1.75 (1.46, 2.10)	1.42 (1.18, 1.72)
‘Mother Only’ vs ‘Neither’	2.55 (2.23, 2.91)	1.33 (1.13, 1.56)
‘Mother and Grandmother’ vs ‘Neither’	3.08 (2.67, 3.56)	1.37 (1.15, 1.64)
‘Mother Only’ vs ‘Grandmother Only’	1.46 (1.22, 1.74)	0.93 (0.76, 1.14)
‘Mother and Grandmother’ vs ‘Grandmother Only’	1.76 (1.47, 2.12)	0.96 (0.78, 1.18)
‘Mother and Grandmother’ vs ‘Mother Only’	1.21 (1.06, 1.39)	1.03 (0.90, 1.19)

^a^ Adjusted for income quintile and location (urban/rural) of neighborhood at birth of mother, mother’s birth year, income quintile and location (urban/rural) of neighborhood at birth of child, child’s birth year, child’s sex, child’s birth order, child’s birth weight, child’s gestational age, child diagnoses before 5^th^ birthday: ADHD, conduct disorder, asthma, injury hospitalization, parent(s) received welfare

### Additional analysis

This paper’s objective was to examine the relationship between age at first birth among mothers and grandmothers and a child’s school readiness. Age at first birth has important implications for the education and employment of mothers.[52] However, not all children born to women who had their first child before age 20 were born when their mother was still an adolescent. Of the 5,179 children whose mothers had their first child before age 20, the mothers of 3,021 (58.3%) were no longer adolescents at the birth of this child (these children were not first-born). To examine whether a history of adolescent motherhood effects children born after age 20, we compared the outcomes of children not born to adolescent mothers with a) children born to mothers who had their first child before age 20 and were born before their mother turned 20, and b) children born to mothers who had their first child before age 20 and were born after their mother turned 20.

The unadjusted analysis showed that, regardless whether the mother was younger or older than 20 when the child was born, children born to mothers having their first child before age 20 had significantly greater odds of not being ready for school than children whose mother was at least 20 when she had her first child (Table 6). After adjusting for a range of covariates, children whose mothers were less than 20 when they had their first child but were at least 20 when they were born were had similar odds of being not ready for school than children not born to adolescent mothers.

**Table 6:** Unadjusted, and adjusted odds ratios of school readiness by Mother’s Adolescent Motherhood Status and Mother’s Age at Birth of Child.

Not Ready for School	Unadjusted OR (95% CI)	Adjusted^a^ OR (95% CI)
Overall		
Mother was not an adolescent mother	Reference	Reference
Mother was an adolescent mother, child born before mother’s 20th birthday	2.08 (1.90, 2.28)	1.26 (1.12, 1.43)
Mother was an adolescent mother, child born after mother’s 20th birthday’	2.16 (1.95, 2.39)	1.04 (0.90, 1.20)
Physical Well-Being		
Mother was not an adolescent mother	Reference	Reference
Mother was an adolescent mother, child born before mother’s 20th birthday	1.92 (1.71, 2.16)	1.21 (1.04, 1.42)
Mother was an adolescent mother, child born after mother’s 20th birthday’	2.16 (1.90, 2.45)	1.11 (0.92, 1.32)
Social Competence		
Mother was not an adolescent mother	Reference	Reference
Mother was an adolescent mother, child born before mother’s 20th birthday	1.90 (1.68, 2.14)	1.30 (1.11, 1.53)
Mother was an adolescent mother, child born after mother’s 20th birthday’	2.00 (1.76, 2.28)	1.01 (0.84, 1.21)
Communication and General Knowledge		
Mother was not an adolescent mother	Reference	Reference
Mother was an adolescent mother, child born before mother’s 20th birthday	1.73 (1.51, 1.98)	1.04 (0.87, 1.24)
Mother was an adolescent mother, child born after mother’s 20th birthday’	2.28 (1.97, 2.63)	0.93 (0.76, 1.14)
Emotional Maturity		
Mother was not an adolescent mother	Reference	Reference
Mother was an adolescent mother, child born before mother’s 20th birthday	1.70 (1.50, 1.92)	1.25 (1.06, 1.47)
Mother was an adolescent mother, child born after mother’s 20th birthday’	1.61 (1.40, 1.84)	0.95 (0.78, 1.15)
Language and Cognitive Development		
Mother was not an adolescent mother	Reference	Reference
Mother was an adolescent mother, child born before mother’s 20th birthday	2.33 (2.06, 2.63)	1.29 (1.10, 1.51)
Mother was an adolescent mother, child born after mother’s 20th birthday’	2.48 (2.17, 2.83)	1.08 (0.90, 1.30)

^a^ Adjusted for income quintile and location (urban/rural) of neighborhood at birth of mother, mother’s birth year, income quintile and location (urban/rural) of neighborhood at birth of child, child’s birth year, child’s sex, child’s birth order, child’s birth weight, child’s gestational age, child diagnoses before 5^th^ birthday: ADHD, conduct disorder, asthma, injury hospitalization, parent(s) received welfare

## Discussion

### Summary of primary findings

The rich Manitoba administrative data allowed examining the multigenerational effect of adolescent motherhood on school readiness. We found that if a woman had her first child during adolescence, her grandchildren tended to be less ready for school. This relationship between a woman and her grandchildren persisted even when the child’s own mother was not an adolescent mother.

### Comparison with other findings

Our findings that children of adolescent mothers are less ready for school are consistent with earlier findings not using the EDI. Children with adolescent mothers lagged behind on physical well-being, social competence, and language and cognitive development. One previous study identified children of teenage mothers to have lower cognitive attainment and proficiency scores, and lower levels of self-control in kindergarten compared with children born to mothers age 20–21, while another showed children born to adolescent mothers to have lower reading, math, and general knowledge scores in kindergarten.[53,54] These outcomes have been attributed to lower educational attainment among adolescent mothers, resulting in less enriching home environments for children, including reading and family activities.[55] Our findings that among children born to mothers whose first child was born before the mother was 20, those born after the mother had turned 20 fared better than children born before their mother had turned 20 is consistent with previous research.[56]

Little research has documented the connections between a grandparent’s adolescent outcomes and the outcomes of their grandchildren. One study found children to have significantly lower math and reading scores if both of their maternal grandparents did not finish high school. This was attributed to educational attainment being a stable measure of socioeconomic status and a heritable component of cognitive achievement.[57] Although not measuring the effects of adolescent motherhood specifically, another paper found that living in poor neighborhoods can have multigenerational effects on cognitive development.[28] Living in poverty results in some of the most significant intergenerational consequences, and adolescent mothers are much more likely to be living in poverty.[27] Even if daughters of adolescent mothers do not become adolescent mothers themselves, they more likely to also live in poverty, which can affect their child’s development.[58] Our findings that grandchildren of adolescent mothers are less ready for school (even if their mothers were not adolescent mothers) fit well with previous findings on the multigenerational effects of poverty.

### Strengths and limitations

Our use of linkable population-based administrative databases has some significant strengths, including large sample size, minimal attrition, the availability of many predictors over several years, and the ability to identify familial relationships. Several limitations should be noted. While accounting for the income quintile of the neighborhood the grandmother lived in when the mother was born, the income quintile of the neighborhood the mother lived in when the child was born, and whether the child was a dependent of someone receiving welfare before the child’s fifth birthday, we could not specify individual income. Mother and grandmother’s educational attainment and marital status could not be accounted for; this could also affect the child’s school readiness. Adolescent mothers are much less likely to finish high school and to live in low income neighborhoods; our adjustment for neighborhood-level income is likely to be capturing most of the differences in educational achievement among adolescent mothers. Since information on grandmother’s age at first birth was only available for mothers born in 1979 or later, our inclusion of many young mothers (47% were adolescent mothers) is not representative of the population. These findings should be replicated in a setting where older mothers are more accurately represented. Despite these limitations, this study was the first to examine the association between intergenerational adolescent motherhood and school readiness.

### Implications for policy, practice and further research

Public education is intended, among other things, to reduce the inequalities of poverty and circumstance. Differences in school readiness can accumulate over time and result in much worse long-term outcomes.[3] Children and grandchildren of adolescent mothers were identified to be at a particular disadvantage as they enter school. To address developmental outcomes for children with a family history of adolescent motherhood, both social and structural interventions are required. Identified as a mechanism for decreased school readiness among children of adolescent mothers, poverty can affect early childhood development in several ways. One of the outcomes of poverty is food insecurity, which has significant implications for child development.[59] To improve health and developmental outcomes, supplementary nutrition should be provided to adolescent mothers (starting in pregnancy) and their children.[58] Unconditional prenatal income supplements, often used to address food insecurity, have resulted in significantly improved birth outcomes for children of low income mothers.[60] Lower levels of school completion among adolescent mothers also contribute to poverty. While some adolescent mothers become pregnant after dropping out of school, those who become pregnant while in school are at higher risk for dropping out.[12] Lack of childcare and other supports often prevent adolescent mothers from returning to school.[61] A reduction in absenteeism and dropout rates has been found among adolescents receiving school-based prenatal care, parent support programs, and school-based child care.[62,63] Investing resources and providing supports to adolescent mothers to improve their economic and social resources should improve not only her children’s school readiness but also her future grandchild’s school readiness.

To our knowledge, this is the first study to examine the multigenerational effects of adolescent motherhood on school readiness. This work also highlights the benefit of multigenerational data linkage across health and social databases. While children and grandchildren of adolescent mothers have been identified to be less ready for school, the mechanisms underlying this multigenerational effect are unclear. The potential pathways that result in these findings should be investigated to better identify the most efficient interventions to address school readiness in this population. Future research examining the economic costs of adolescent motherhood should not only consider the costs to the mother and the child, but also include the costs of adolescent motherhood for grandchildren.

## Conclusions

The social consequences and economic costs of adolescent childbearing have been examined in many contexts, with poorer outcomes of mothers and children being attributed to a series of disadvantages (economic, social supports, etc.) that these young mothers face.[64] When calculating the costs and consequences of adolescent pregnancy, the potential multigenerational implications of adolescent motherhood have not been considered. Our findings imply that adolescent childbearing has significant implications for early childhood development—not just for the child of that mother, but also for the grandchild of that mother. Interventions to improve outcomes of children born to adolescent mothers should also include grandchildren of adolescent mothers.

## Supporting information

S1 Fig Cohort selection. (DOCX)

S1 File Defining childhood covariates. (DOCX)

S1 Table Definitions of childhood covariates between birth and age 5. (DOCX)

S2 Table Results of adjusted logistic regression models for school readiness by grandmother’s adolescent motherhood status (n = 14,298). (DOCX)

S3 Table Results of adjusted logistic regression models for school readiness by mother’s adolescent motherhood status (n = 14,298). (DOCX)

S4 Table Results of adjusted logistic regression models for school readiness by intergenerational adolescent motherhood status (n = 14,298). (DOCX)
