# Supplementary figures and images for: The multigenerational effects of adolescent motherhood on school readiness: A population-based retrospective cohort study
Source: PLoS One. 2019 Feb 6;14(2):e0211284. doi: 10.1371/journal.pone.0211284 (PMC6364914; doi:10.1371/journal.pone.0211284)

*S1 Figure. Cohort Formation*

**
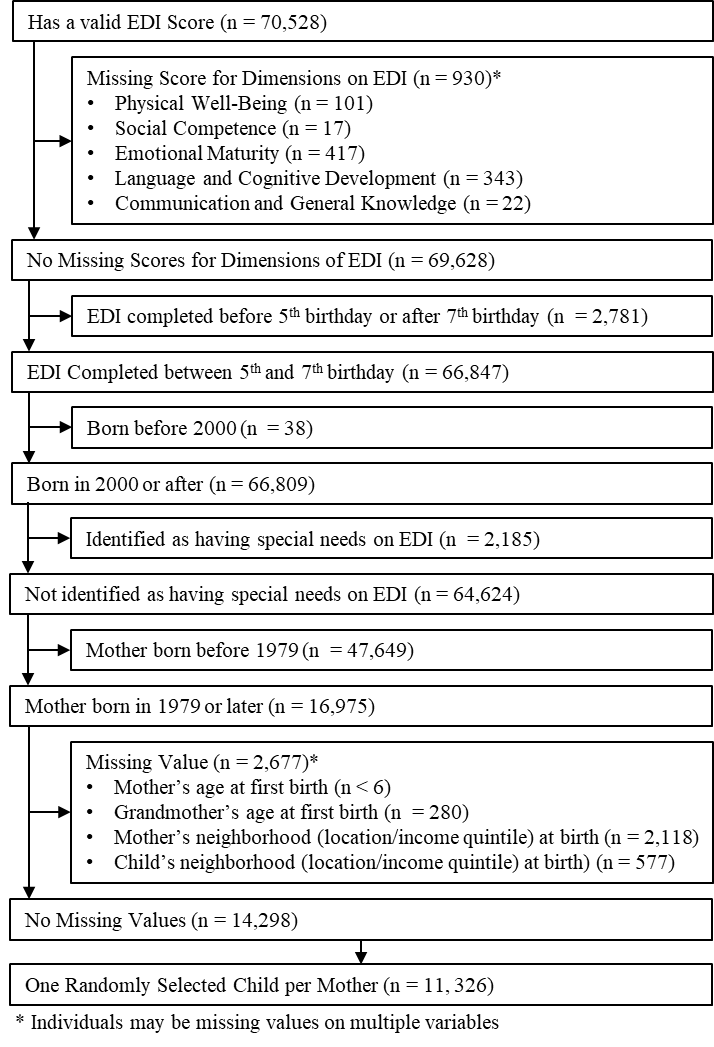
**

Supplement: S1 Fig — (DOCX) [file pone.0211284.s001.docx]
