# Supplementary material for: The multigenerational effects of adolescent motherhood on school readiness: A population-based retrospective cohort study
Source: PLoS One. 2019 Feb 6;14(2):e0211284. doi: 10.1371/journal.pone.0211284 (PMC6364914; doi:10.1371/journal.pone.0211284)
Supplement: S1 File — (DOCX) [file pone.0211284.s002.docx]

**S1 File. Defining Childhood Covariates**

Health is defined by physician claims (International Classification of Diseases, Ninth Revision, Clinical Modification [ICD-9-CM] codes), hospital discharge abstracts (ICD-9-CM codes before 2004, International Classification of Diseases, Ninth Revision, Canada [ICD-10-CA] codes in 2004 and later), and prescriptions (using Anatomical Therapeutic Chemical classification system codes).
