# Supplementary material for: The multigenerational effects of adolescent motherhood on school readiness: A population-based retrospective cohort study
Source: PLoS One. 2019 Feb 6;14(2):e0211284. doi: 10.1371/journal.pone.0211284 (PMC6364914; doi:10.1371/journal.pone.0211284)
Supplement: S1 Table — (DOCX) [file pone.0211284.s003.docx]

**S1 Table.** Definitions of Childhood Covariates between Birth and Age 5

| **Term** | **Definition** |
| --- | --- |
| ADHD | At least one hospitalization with a diagnosis of hyperkinetic syndrome (ICD-9-CM code 314; ICD-10-CA code F90), at least one physician visit with a diagnosis of hyperkinetic syndrome (ICD-9-CM code 314), or two or more prescriptions for psychostimulant medication without a corresponding diagnosis of conduct disorder or narcolepsy (ICD-9-CM codes 312, 347; ICD-10-CA code F91, G47) before a child’s fifth birthday.(1) |
| Conduct disorder | At least one physician visit with a diagnosis of conduct disorder (ICD-9-CM code 312), or one hospitalization with a diagnosis of conduct disorder (ICD-9-CM code 312; ICD-10-CA code F91 except F91.3) before a child’s fifth birthday.(2) |
| Asthma | At least one physician visit with a diagnosis of asthma (ICD-9-CM code 493), or one hospitalization with a diagnosis of asthma (ICD-9-CM code 493; ICD-10-CA code J45) before a child’s fifth birthday.(3) |
| Injuries | At least one hospitalization with an external cause of injury code (ICD-9-CM codes E800-E869, E880-E929; E950-E978, E990-E999; ICD-10-CA V01-X60-Y09; Y10-Y36, Y870-Y872, Y890-Y891, Y899, Y90, Y91).(4) |
| Welfare | Being the dependent of someone who had at least two consecutive months of EIA receipt before age five.(5) |

1. Manitoba Centre for Health Policy. Concept: Attention-Deficit Hyperactivity Disorder (ADHD). 2015.

2. Manitoba Centre for Health Policy. Concept: Conduct Disorder [Internet]. 2016 [cited 2017 Nov 2]. Available from: http://mchp-appserv.cpe.umanitoba.ca/viewConcept.php?conceptID=1449

3. Manitoba Centre for Health Policy. Concept: Asthma - Measuring Prevalence [Internet]. 2012 [cited 2016 Oct 18]. Available from: http://mchp-appserv.cpe.umanitoba.ca/viewConcept.php?conceptID=1305

4. Manitoba Centre for Health Policy. Concept: External Cause of Injury Codes and Injury Categories [Internet]. 2015 [cited 2018 May 28]. Available from: http://mchp-appserv.cpe.umanitoba.ca/viewConcept.php?conceptID=1168

5. Wall-Wieler E, Roos L, Chateau D, Roos N. Social Context of Welfare in Manitoba, Canada. Soc Indic Res. 2016;
