# Supplementary material for: The multigenerational effects of adolescent motherhood on school readiness: A population-based retrospective cohort study
Source: PLoS One. 2019 Feb 6;14(2):e0211284. doi: 10.1371/journal.pone.0211284 (PMC6364914; doi:10.1371/journal.pone.0211284)
Supplement: S4 Table — (DOCX) [file pone.0211284.s006.docx]

**S4 Table.** Results of Adjusted Logistic Regression Models for School Readiness by Intergenerational Adolescent Motherhood Status (n = 14,298)

|  | Not Ready for School | | | | | |
| --- | --- | --- | --- | --- | --- | --- |
|  | Overall | Physical  Well-Being | Social Competence | Communication and General Knowledge | Emotional Maturity | Language and Cognitive Development |
| **Intergenerational Adolescent Motherhood** |  |  |  |  |  |  |
| Neither | Reference | Reference | Reference | Reference | Reference | Reference |
| Grandmother Only | 1.34 (1.19, 1.52) | 1.35 (1.15, 1.59) | 1.44 (1.22, 1.69) | 1.40 (1.17, 1.68) | 1.20 (1.01, 1.42) | 1.34 (1.13, 1.59) |
| Mother Only | 1.25 (1.12, 1.39) | 1.25 (1.09, 1.44) | 1.30 (1.12, 1.50) | 1.13 (0.96, 1.32) | 1.16 (1.00, 1.34) | 1.33 (1.15, 1.53) |
| Mother and Grandmother | 1.29 (1.14, 1.46) | 1.33 (1.15, 1.55) | 1.35 (1.16, 1.58) | 1.08 (0.91, 1.28) | 1.18 (1.01, 1.39) | 1.34 (1.15, 1.57) |
| **Mother Variables at Birth** |  |  |  |  |  |  |
| Urban Neighborhood | 0.87 (0.80, 0.95) | 0.84 (0.75, 0.93) | 0.87 (0.78, 0.98) | 0.65 (0.57, 0.74) | 0.88 (0.78, 1.00) | 0.80 (0.71, 0.90) |
| Income Quintile of Neighborhood |  |  |  |  |  |  |
| 1 (Lowest) | 1.43 (1.25, 1.63) | 1.17 (0.99, 1.37) | 1.31 (1.10, 1.56) | 1.74 (1.42, 2.12) | 1.05 (0.88, 1.24) | 1.40 (1.18, 1.67) |
| 2 | 1.08 (0.94, 1.24) | 0.97 (0.81, 1.15) | 1.17 (0.97, 1.41) | 1.39 (1.12, 1.71) | 0.98 (0.81, 1.17) | 1.16 (0.96, 1.39) |
| 3 | 0.98 (0.85, 1.13) | 0.82 (0.68, 0.98) | 1.12 (0.92, 1.26) | 1.26 (1.01, 1.57) | 0.97 (0.80, 1.17) | 1.00 (0.82, 1.22) |
| 4 | 0.88 (0.76, 1.02) | 0.77 (0.63, 0.93) | 0.90 (0.73, 1.10) | 1.03 (0.82, 1.30) | 0.86 (0.71, 1.05) | 0.80 (0.65, 0.99) |
| 5 (Highest) | Reference | Reference | Reference | Reference | Reference | Reference |
| Year |  |  |  |  |  |  |
| 1979-1984 | 0.43 (0.31, 0.59) | 0.56 (0.38, 0.81) | 0.38 (0.27, 0.56) | 0.42 (0.27, 0.64) | 0.47 (0.32, 0.70) | 0.30 (0.21, 0.44) |
| 1985-1991 | 0.62 (0.46, 0.84) | 0.76 (0.53, 1.09) | 0.53 (0.38, 0.76) | 0.65 (0.44, 0.98) | 0.63 (0.43, 0.91) | 0.43 (0.31, 0.60) |
| 1992-1997 | Reference | Reference | Reference | Reference | Reference | Reference |
| **Child Variables at Birth** |  |  |  |  |  |  |
| Urban Neighborhood | 1.03 (0.94, 1.13) | 1.06 (0.94, 1.18) | 1.06 (0.94, 1.19) | 1.02 (0.90, 1.16) | 1.06 (0.94, 1.20) | 0.96 (0.86, 1.08) |
| Income Quintile of Neighborhood |  |  |  |  |  |  |
| 1 (Lowest) | 1.33 (1.15, 1.54) | 1.25 (1.03, 1.51) | 1.49 (1.22, 1.83) | 1.20 (0.97, 1.49) | 1.06 (0.94, 1.20) | 1.53 (1.25, 1.88) |
| 2 | 1.16 (1.00, 1.35) | 1.18 (0.97, 1.44) | 1.23 (1.00, 1.52) | 0.99 (0.79, 1.25) | 1.34 (1.10, 1.65) | 1.21 (0.98, 1.50) |
| 3 | 1.16 (1.00, 1.35) | 1.16 (0.95, 1.42) | 1.27 (1.03, 1.57) | 1.05 (0.83, 1.32) | 1.30 (1.06, 1.60) | 1.20 (0.97, 1.49) |
| 4 | 1.17 (1.00, 1.36) | 1.08 (0.87, 1.33) | 1.28 (1.03, 1.57) | 1.09 (0.86, 1.39) | 1.18 (0.96, 1.46) | 1.18 (0.95, 1.49) |
| 5 (Highest) | Reference | Reference | Reference | Reference | Reference | Reference |
| Year |  |  |  |  |  |  |
| 2000-2003 | 1.44 (1.27, 1.62) | 1.19 (1.02, 1.39) | 1.21 (1.03, 1.43) | 1.50 (1.25, 1.79) | 1.34 (1.14, 1.58) | 1.51 (1.28, 1.77) |
| 2004-2007 | 1.23 (1.12, 1.34) | 1.18 (1.05, 1.32) | 1.35 (1.20, 1.52) | 1.19 (1.04, 1.36) | 1.23 (1.09, 1.39) | 1.28 (1.13, 1.44) |
| 2008-2010 | Reference | Reference | Reference | Reference | Reference | Reference |
| Sex |  |  |  |  |  |  |
| Female | Reference | Reference | Reference | Reference | Reference | Reference |
| Male | 2.05 (1.91, 2.21) | 1.63 (1.47, 1.77) | 2.22 (2.01, 2.45) | 2.01 (1.80, 2.24) | 2.88 (2.59, 3.20) | 1.86 (1.69, 2.05) |
| Birth Order | 1.26 (1.20, 1.33) | 1.26 (1.18, 1.33) | 1.22 (1.14, 1.30) | 1.41 (1.32, 1.51) | 1.16 (1.09, 1.23) | 1.27 (1.20, 1.35) |
| Low Birth Weight | 1.11 (0.91, 1.35) | 1.07 (0.84, 1.36) | 0.96 (0.74, 1.23) | 1.14 (0.87, 1.50) | 1.02 (0.79, 1.32) | 1.18 (0.92, 1.51) |
| Preterm | 1.08 (0.92, 1.29) | 1.14 (0.93, 1.40) | 1.21 (0.98, 1.50) | 1.10 (0.87, 1.39) | 1.14 (0.91, 1.41) | 1.15 (0.93, 1.42) |
| **Child Variables Between Birth and Age 5** |  |  |  |  |  |  |
| ADHD Diagnosis | 2.01 (1.55, 2.59) | 1.63 (1.23, 2.15) | 2.73 (2.10, 3.55) | 1.44 (1.04, 1.98) | 2.74 (2.10, 3.56) | 1.64 (1.23, 2.18) |
| Conduct Disorder Diagnosis | 1.38 (1.09, 1.74) | 1.09 (0.82, 1.44) | 1.65 (1.27, 2.15) | 1.11 (0.80, 1.53) | 1.84 (1.42, 2.39) | 1.08 (0.81, 1.44) |
| Hospitalization for Injury | 1.26 (0.89, 1.76) | 0.98 (0.65, 1.48) | 1.31 (0.88, 1.94) | 1.02 (0.65, 1.60) | 1.40 (0.94, 2.09) | 1.09 (0.73, 1.63) |
| Asthma Diagnosis | 1.07 (0.98, 1.16) | 1.01 (0.91, 1.13) | 1.01 (0.90, 1.13) | 0.96 (0.84, 1.08) | 1.06 (0.94, 1.18) | 1.00 (0.89, 1.12) |
| Parent(s) Received Welfare | 1.77 (1.63, 1.92) | 2.02 (1.82, 2.24) | 1.47 (1.32, 1.64) | 1.68 (0.49, 1.89) | 1.39 (1.25, 1.55) | 1.99 (1.79, 2.22) |
| C-Statistic | 0.694 | 0.685 | 0.689 | 0.697 | 0.689 | 0.712 |
